# Mesophyll protoplasts: a key tool for deciphering plant nutrient starvation pathways

**DOI:** 10.3389/fpls.2026.1825102

**Published:** 2026-07-23

**Authors:** Tian Yashan, She Jianju, Lin Jinhui, Chen Feng, Song Zhuoran, Liu Jiayi, He Yan, Ma Junhan, Cheng Guoting, Wang Yanfeng, Dang Fengfeng

**Affiliations:** 1 Shaanxi Key Laboratory of Research and Utilization of Resource Plants on the Loess Plateau, College of Life Sciences, Yan’an University, Yan’an, Shaanxi, China; 2 Fruit Research Institute, Fujian Academy of Agricultural Sciences, Fuzhou, China

**Keywords:** Fe starvation, fit, mesophyll protoplasts, Pi starvation, SPX1

## Abstract

The mesophyll protoplast transient expression system is an essential and robust methodology for examining gene expression regulation. Despite its potential, it has not been effectively employed to elucidate the genetic regulatory pathways and networks underlying plant responses to nutrient starvation, such as those involving phosphorus (Pi) and iron (Fe). In this study, we identified differentially expressed genes in response to Pi starvation in *Arabidopsis* using transcriptome analysis and RT-qPCR validation. Our findings revealed that Pi starvation significantly upregulated the expression of Pi-starvation induced (*PSI*) genes, including *SPX1*, *SPX3*, *IPS1*, and *PS2*, while simultaneously downregulating the expression of Fe starvation-responsive genes, such as *FIT*, *IRT1*, and *FRO2*. Additionally, through a dual luciferase transient expression assay in mesophyll protoplasts, we demonstrated that the transcription factor PHR1 serves as a crucial transcriptional regulator, modulating the expression of phosphate starvation response (*PSR*) genes. This regulation significantly enhances the transcriptional activity of the *SPX1*, *SPX2*, *SPX3*, *IPS1*, *PS2*, and *PHT1;4* promoters. Upon the addition of the SPX1 protein, the activation of these gene promoters by PHR1 were alleviated. Concurrently, the transcriptional regulator FIT, which governs the expression of genes responsive to Fe starvation, markedly increased the transcriptional activity of the *IRT1* and *FRO2* promoters. Based on these findings, we propose the mesophyll protoplast transient expression system as a rapid and reliable method for investigating complex genetic networks. Overall, our study provides substantial evidence for understanding the role of the mesophyll protoplast transient expression system in elucidating the genetic regulatory pathways and networks involved in plant responses to nutrient starvation.

## 1 Introduction

Phosphorus (Pi) and iron (Fe) are essential elements for plant growth and development. However, in both natural and agricultural ecosystems, the availability of Pi and Fe is frequently limited by the physicochemical properties of the soil (Lynch and Brown, 2001; Briat et al., 2015). In soils, Pi is susceptible to chemical fixation, which diminishes its availability. For example, in acidic conditions, Pi can be immobilized by Fe^3+^ and Al^3+^ ions, whereas in alkaline conditions, it tends to bind with Ca^2+^ ions, thereby hindering plant absorption (Hinsinger, 2001; Ryan et al., 2001; Kochian et al., 2004; Abel, 2017). To mitigate Pi starvation, plants have evolved a sophisticated regulatory network centered on the SPX1-PHR1 module (Lv et al., 2014; Puga et al., 2014; Wang et al., 2014; Ried et al., 2021). This network detects intracellular Pi levels and modulates the expression of a series of downstream Pi starvation-responsive (*PSR*) genes to maintain Pi homeostasis (Bustos et al., 2010; Paz-Ares et al., 2022). Concurrently, as a cofactor for various critical enzymes and pigments, the uptake and utilization of Fe are also tightly regulated (Gao and Dubos, 2021). Under conditions of Fe starvation, the core transcription factor FIT forms heterodimers with bHLH family proteins bHLH38/39/100/101 in *Arabidopsis*, thereby activating the expression of genes related to Fe uptake, such as *IRT1* and *FRO2* (Yuan et al., 2008; Wang et al., 2013; Li et al., 2023; Gao et al., 2024). Despite advancements in the field, the rapid and reliable identification of key genes, regulatory modules, pathways, and networks remains a critical challenge in plant nutrition starvation research.

Previous studies have identified core regulatory genes and modules, such as SPX1-PHR1 and FIT-*FRO2/IRT1*, through the construction of genetic lines (Wang et al., 2013, 2014; Cui et al., 2018). However, these traditional approaches are hindered by limitations such as lengthy experimental durations, low efficiency, and significant space requirements, which impede their ability to facilitate rapid and stable analyses of gene regulatory pathways and networks. Moreover, the advent of high-throughput sequencing technologies has generated a wealth of gene expression data, necessitating the development of efficient methods to rapidly and accurately pinpoint key regulatory modules and networks from these large datasets in future research (Wang et al., 2009, 2021). Historically, numerous studies have illustrated that the protoplast transient expression system, which involves the extraction of mesophyll cell protoplasts from plant leaves through enzymatic digestion and the introduction of exogenous DNA plasmids into cells via PEG-Ca^2+^-mediated transformation, facilitates the efficient expression of target proteins within a matter of hours. For instance, Abel and Theologis (1994) revealed an improved protocol to isolate and transiently transform mesophyll protoplasts of *Arabidopsis thaliana*. Moreover, Chen K, et al. (2023) summarized the optimization and comparison of protoplast isolation methods for various plants. Offering an efficient, controllable, and stable means to dissect genetic regulatory networks, this system allows for the direct and dynamic examination of regulatory gene effects on downstream promoter activity in a near-physiological cellular context, thereby facilitating the rapid and reliable identification of key regulatory modules and networks (Yoo et al., 2007; Lee et al., 2017; Pasternak et al., 2020; Sahab et al., 2024). Compared to conventional genetic analysis techniques, this method presents notable advantages, including reduced experimental durations, high throughput, and simplified operation.

In this study, we observed that Pi starvation markedly upregulated the expression of a series of Pi-starvation induced (*PSI*) genes, including *SPX1*, *SPX3*, *IPS1*, and *PS2*, among others, while significantly downregulating the expression of Fe starvation-responsive genes such as *FIT*, *IRT1*, and *FRO2*. Utilizing the mesophyll protoplast transient expression system, we further demonstrated that PHR1 substantially enhanced the transcriptional activity of *SPX1*, *SPX2*, *SPX3*, *IPS1*, *PS2*, and *PHT1;4* promoters by approximately 14-, 44-, 28-, 42-, 17-, and 9-fold, respectively. Additionally, the presence of the SPX1 protein was found to inhibit PHR1-mediated activation of these promoters. Moreover, FIT was shown to significantly augment the transcriptional activity of *IRT1* and *FRO2* promoters by approximately 23- and 24- fold, respectively. These results are in strong agreement with previous findings obtained through genetic approaches. Collectively, this study not only provides novel insights into the regulatory pathways governing plant responses to Pi starvation and Pi-Fe interactions but also highlights the significant advantages of the mesophyll cell protoplast transient expression system in elucidating the genetic regulatory networks underlying plant responses to nutrient starvation, emphasizing its stability, rapidity, and high-throughput capacity.

## 2 Materials and methods

### 2.1 Plant materials and growth conditions

In this study, the *Arabidopsis thaliana* ecotype Columbia-0 (Col-0) was employed as the wild type (WT). *Arabidopsis* seeds underwent surface sterilization using 75% ethanol for 1 minute followed by a 5-minute treatment with a 1% sodium hypochlorite solution, and treated with vernalization of 4 °C for 48 hours. After post-vernalization, the seeds were sown on half-strength Murashige and Skoog (MS) medium containing 0.8% agar (Sigma, catalog no. A1296) for a duration of 9 days. Subsequently, the seedlings were transferred to a half-strength MS agarose medium (BIOWEST, Spain) supplemented with either 10 (-Pi) and 625 (+Pi) μM KH_2_PO_4_ for 8 days. Following this treatment, roots were harvested for reverse transcription quantitative PCR (RT-qPCR). For the transient expression assay in *Arabidopsis* mesophyll protoplasts, seeds were rinsed five times with double-distilled water (ddH_2_O), vernalized at 4 °C for 48 hours, and then sown in a steam-sterilized soil mixture composed of peat moss and vermiculite in a 2: 1 volume ratio. Young and fully expanded leaves from 3-week-old plants were subsequently selected for use. *Arabidopsis* seedlings were cultivated at 22 ± 2 °C in an incubator with a maintained relative humidity of 70 ± 5%, under a 16-hour light/8-hour dark photoperiod, with a photosynthetic photon flux density (PPFD) of 100 µmol photons m^−2^ s^−1^.

### 2.2 Molecular cloning and plasmid construction

Effector and reporter vectors for the transient expression assay were constructed utilizing established molecular biology protocols and homologous recombination techniques. For the effector vectors, *Arabidopsis* cDNA served as the template for PCR amplification of the coding sequences (CDS) of *SPX1*, *SPX2*, *PHR1*, and *FIT.* These sequences were subsequently cloned into the *Xba*I*­Spe*I sites of the *p19-GFP* vector, resulting in the formation of the constructs *pCaMV35S-SPX1-GFP*, *pCaMV35S-SPX2-GFP*, *pCaMV35S-PHR1-GFP*, and *pCaMV35S-FIT-GFP*. For the reporter vectors, promoter fragments of *ISP1*, *SPX1*, *IRT1, FRO2* and other genes were amplified via PCR using *Arabidopsis* genomic DNA as the template. These fragments were then cloned into the *Hind*III­*BamH*I sites of the *pGreen*­*0800*­*LUC* vector, yielding the *pSPX1:LUC*, *pISP1:LUC*, *pIRT1:LUC*, *pFRO2:LUC*, and other reporter constructs. The primers utilized for the generation of these vectors are specified in **Supplementary Table 1**.

### 2.3 Extraction and purification of plasmid

Plasmids were extracted and purified from *Escherichia coli* DH5α transformed cultures using the Endofree Maxi Plasmid Kit (DP117, TIANGEN, Beijing, China), following the manufacturer’s instructions. In brief, the effector (such as *pCaMV35S-SPX1-GFP*, *pCaMV35S-SPX2-GFP*, *pCaMV35S-PHR1-GFP*, and *pCaMV35S-FIT-GFP*) and reporter (such as *pISP1:LUC*, *pSPX1:LUC*, *pIRT1:LUC*, *pFRO2:LUC*, and so on) plasmids were transformed into *E.coli* DH5α competent cells and plated on LB agar. The cultures were incubated overnight. Subsequently, the bacterial colonies were inoculated into 200 mL of Terrific Broth (TB) medium supplemented with either 75 μg mL^-1^ ampicillin or 50 μg mL^-1^ kanamycin and incubated overnight at 37 °C with shaking at 200 rpm. Plasmids were then harvested and extracted using the Endofree Maxi Plasmid Kit (DP117). The plasmids were precipitated with isopropanol and 5 M NaCl, washed twice with 70% ethanol, and dissolved in ultrapure distilled water (Thermo Fisher Scientific Product No.: 10977035). The concentration of the plasmids was adjusted to 2000 ng µL^-1^.

### 2.4 Protoplast isolation

Protoplast isolation was conducted following a previous protocol, with minor modifications (Yoo et al., 2007). In brief, the twenty youngest leaves of *Arabidopsis* seedlings were harvested, and the basal and marginal sections were excised. The selected leaf segments were then precisely cut into approximately 1 mm strips and immediately immersed in 10 mL of an enzyme solution. This solution comprised 1.5% (w/v) cellulase R10, 0.3% (w/v) macerozyme R10, 0.4 M mannitol, 20 mM KCl, 20 mM MES (pH 5.7), and 10 mM CaCl_2_. The samples were subsequently subjected to vacuum infiltration in darkness for 30 minutes, followed by enzymatic digestion at room temperature for 4 hours under dark conditions with gentle agitation at approximately 30 rpm.

### 2.5 Protoplast purification

Following infiltration of a 70 µm cell strainer with W5 solution, which comprising 1–154 mM NaCl, 125 mM CaCl_2_, 5 mM KCl, 1.5 mM MES (pH 5.7), and 5 mM Glucose, the enzymatic digestion process was halted by the addition of 10 mL of W5 solution. The digestion mixture was subsequently collected through a 70 µm cell strainer. Protoplast cells were then isolated via centrifugation at 180 g and 4 °C for 2 minutes. These cells were re-suspended and washed with 10 mL of W5 solution on ice for 30 minutes. Purified protoplast cells were obtained by carefully removing the W5 solution. The cells were then re-suspended in MMG solution, which contained 15 mM MgCl_2_, 5.2 mM MES (pH 5.7), and 0.4 M mannitol, and their density was adjusted to 1×10^5^ cells ml^-1^ using a Hemocytometer (Neubauer, Marienfeld, Germany).

### 2.6 Protoplast transfection

The transfection of protoplast cells was conducted as previously described (Yoo et al., 2007). In brief, 20 μg of the effector plasmid and 20 μg of the reporter plasmid were added to 220 µL of protoplast cells. Subsequently, 220 µL of freshly prepared PEG-Ca^2+^ solution (40% PEG4000, 200 mM mannitol, and 100 mM CaCl_2_) was immediately added and mixed gently and rapidly. The mixture was incubated for 6 minutes at room temperature. The incubation was promptly terminated by adding 880 µL of W5 solution, followed by centrifugation at 180 g and 4 °C for 2 minutes to collect the protoplast cells, which were then re-suspended and washed with 1 mL of W5 solution. The transformed protoplast cells were subsequently harvested and gently re-suspended in 1 mL of WI solution, containing 0.5 M mannitol, 4 mM MES (pH 5.7), and 20 mM KCl. The WI solution containing the protoplast cells was then transferred to a 12-well culture plate and incubated at 25 °C in the dark. After a 10-hour incubation, the protoplast cells were collected by centrifugation, and the transformation efficiency was assessed by analyzing green fluorescent protein (GFP) expression using fluorescence microscopy.

### 2.7 Reagent information

Cellulase R-10 and Macerozyme R-10 enzymes were procured from Yakult Honsha, located in Tokyo, Japan. The following reagents were sourced from Sangon Biotech in Shanghai, China: D-Mannitol (Order NO. A100122), PEG4000 (Order NO. A620431), KCl (Order NO. A100395), MES (Order NO. A100169), NaCl (Order NO. A100241), and MgCl_2_·6H_2_O (Order NO. A631017). Additionally, CaCl_2_·2H_2_O (Order NO. 223506) was obtained from Sigma-Aldrich in the United States.

### 2.8 Dual-luciferase reporter assay

A transfection efficiency exceeding 70% was attained, and a dual-luciferase reporter assay was subsequently conducted. Briefly, protoplast cells were lysed using a cell lysate solution through vigorous agitation, followed by the collection of the supernatant via centrifugation at 10000 g for 5 minutes. The activities of firefly luciferase (LUC) and Renilla luciferase (REN) were then assessed using a dual-luciferase reporter gene assay kit (Yeasen, Shanghai, China) in accordance with the manufacturer’s instructions, employing the Dual-Glo^®^ Luciferase Assay System (Promega, USA). The relative promoter activity was quantified as the ratio of *LU*C expression, driven by the reporter gene promoter, to *REN* expression under the control of the *CaMV35S* promoter.

### 2.9 Expression analysis

Total RNA was extracted from *Arabidopsis thaliana* roots utilizing the RNAprep Pure Plant Kit (Tiangen Biotech, Beijing, China). Subsequently, 500 ng of RNA served as a template for cDNA synthesis, performed with the TaKaRa PrimeScript RT-PCR Kit (TaKaRa) in accordance with the manufacturer’s protocol. Quantification of gene expression levels was conducted using a Light-Cycler 480 Real-Time PCR System (Roche, Switzerland) with ChamQ SYBR qPCR Master Mix (Vazyme), employing specific primers detailed in **Supplementary Table 1**. The expression levels were normalized to *UBQ10*. Additionally, Using RNA-seq data from previous studies (Wang et al., 2025), we obtained expression levels of genes significantly upregulated or downregulated under Pi starvation, represented as FPKM (Fragments Per Kilobase of exon model per Million mapped reads). Then, we used TBtools software to create a heatmap of the Log_2_-transformed values for these target genes (Chen C, et al., 2023).

### 2.10 Statistical analysis

Statistically significant differences between groups were evaluated using Student’s *t*-test, with asterisks denoting significant differences (**, *P<0.01*) or different letters (uppercase letters, *P* < *0.01*, Tukey’s multiple comparisons test) in the figures. Each experiment included a minimum of three biological replicates, which were analyzed independently. All values are presented as means ± standard error (SE).

### 2.11 Accession numbers

Sequence data pertinent to this study are available on the *Arabidopsis* Information Resource (TAIR, https://www.arabidopsis.org/) under the following identifiers: *PHR1* (AT4G28610), *SPX1* (AT5G20150), *SPX2* (AT2G26660), *SPX3* (AT2G45130), *IPS1* (AT3G09922), *AT4* (AT5G03545), *PS2* (AT1G73010), *PHT1;4* (AT2G38940), *PHT1;5* (AT2G32830), *PHT1;8* (AT1G20860), *PHT1;9* (AT1G76430), *FIT* (AT2G28160), *IRT1* (AT4G19690), *IRT2* (AT4G19680), *NAS1* (AT5G04950), NA*S2* (AT5G56080), *bHLH38* (AT3G56970), *bHLH39* (AT3G56980), *bHLH100* (AT2G41240), *bHLH101* (AT5G04150), and *UBQ10* (AT4G05320).

## 3 Results

### 3.1 A rapid, reliable, and throughout system of *Arabidopsis* mesophyll protoplast in transient expression

**Figure 1:**
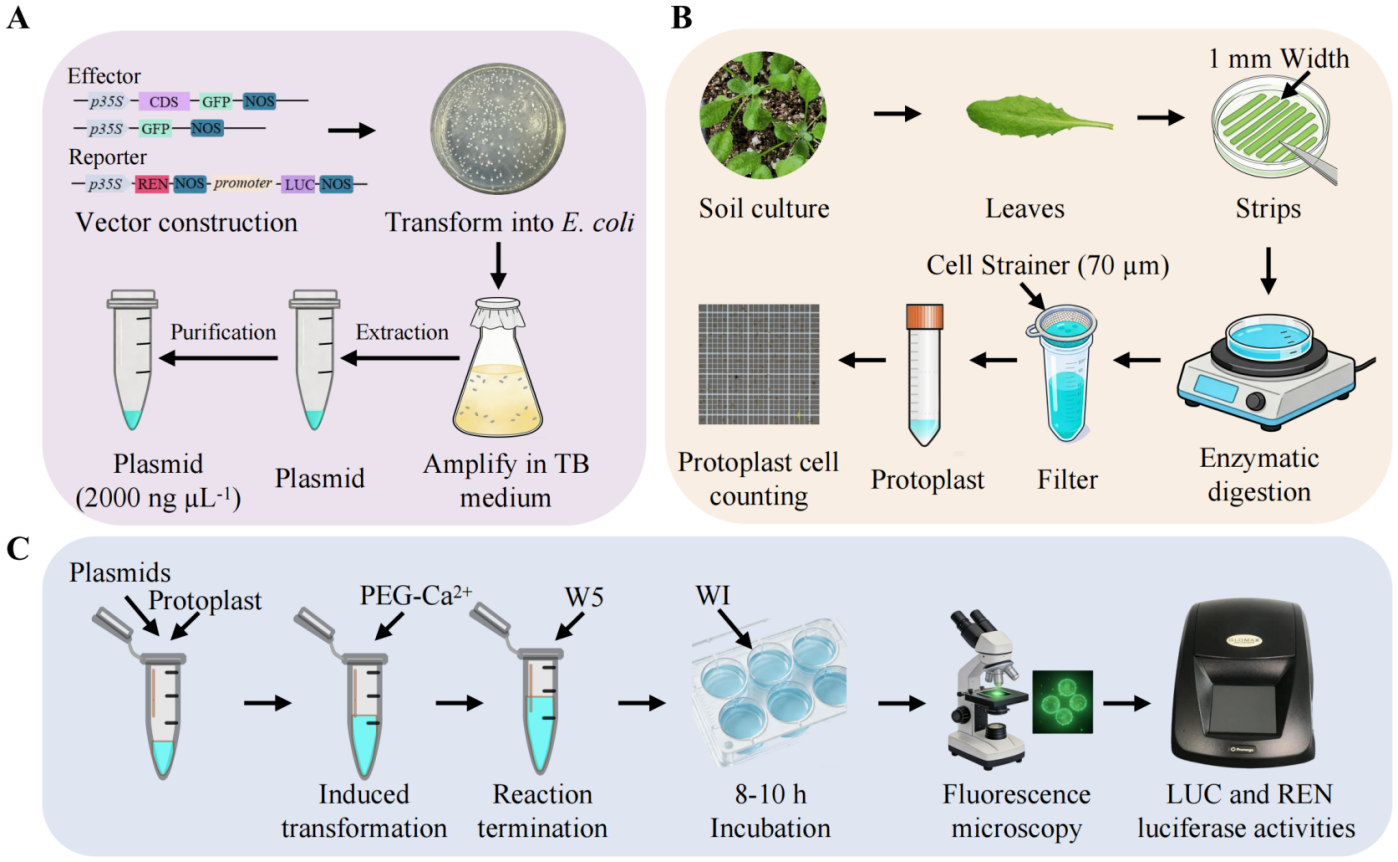
The visual flow-process diagram delineates the *Arabidopsis* mesophyll protoplast transient expression system. **(A)** Schematic diagram representation of the isolation and purification processes for effector and reporter plasmids utilized in the dual-luciferase reporter assay. **(B)** Schematic procedure for the preparation of *Arabidopsis* mesophyll cell protoplasts. **(C)** Schematic diagram the transient expression system employing DNA plasmids in protoplasts for the dual-luciferase reporter assay.

Numerous studies have demonstrated that protoplast and tobacco leaf transient expression systems, particularly protoplasts, possess significant potential for applications in investigating interactions between transcription factors and promoters, protein-protein interactions, and signal transduction pathways (Yang et al., 2000; Sparkes et al., 2006; Yoo et al., 2007; Son et al., 2021; Higa et al., 2025; Fulcher et al., 2026). To further explore and substantiate the utility of transient expression systems, we conducted a rapid and high-throughput investigation into the molecular relationships among genes involved in plant responses to Pi starvation. This was achieved through a series of procedures, including the large-scale extraction and purification of effector and reporter plasmids, preparation of *Arabidopsis* mesophyll cell protoplasts, PEG-Ca^2+^-mediated transformation, analysis of fusion protein expression, and quantitative analysis of reporter genes. In this study, we specifically cloned the coding sequences of target proteins into a reporter vector containing a GFP tag, which was driven by the constitutive *CaMV35S* promoter. Additionally, we cloned the promoters of target genes into an effector vector containing *REN* and *LUC* reporter genes. We then performed large-scale extraction and purification of both effector and reporter plasmids. Importantly, plasmids at a concentration of 2000 ng µL^-1^ were utilized for subsequent transient expression experiments (**Figure 1A**). Protoplasts isolated from the leaves of 3-week-old *Arabidopsis* plants were subsequently prepared, of which 1×10^5^ cells were employed for the dual-luciferase reporter assay (**Figure 1B**). Furthermore, the prepared protoplasts were used for expression analysis with various combinations of effector and reporter plasmids, as well as for exploring molecular interactions among genes. Notably, approximately 80 combinations of transient expression experiments, each with three biological replicates, could be conducted using 50 milliliters of protoplast cells (**Figure 1C**). In conclusion, the *Arabidopsis* mesophyll cell protoplast fusion protein expression system offers a rapid, reliable, and high-throughput approach for investigating molecular interactions among genes.

### 3.2 Screening of Pi-starvation induced genes and Pi-starvation suppressed genes in *Arabidopsis*

**Figure 2:**
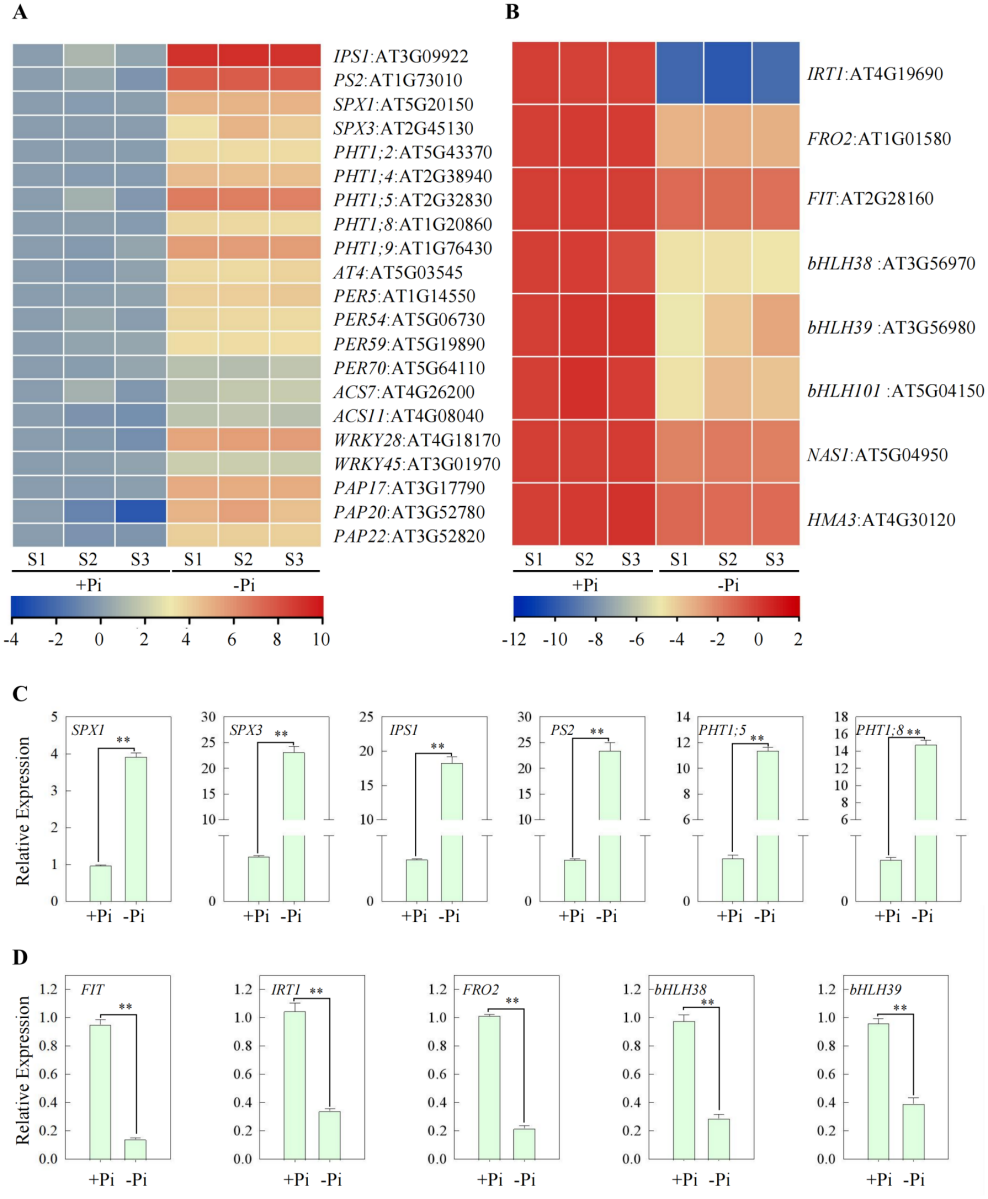
Pi starvation significantly induced Pi starvation-responsive genes expression and inhibited Fe starvation-responsive genes expression. **(A)** A heat map shows a series of Pi starvation-responsive genes significantly induced by Pi starvation. **(B)** A heat map shows a series of Fe starvation-responsive genes significantly inhibited by Pi starvation. **(C)** RT-qPCR analysis of Pi starvation-responsive genes transcript levels. **(D)** RT-qPCR analysis of Fe starvation-responsive genes transcript levels. WT seedlings were treated under Pi starvation for 8 days, and the differentially expressed genes were analyzed by RT-qPCR. Data are presented as the means ± SE with a sample size of *n* = 3. Asterisks denote statistically significant differences as determined by Student’s t-test (**P<0.01).

Utilizing this transient expression system, we explored the genetic regulatory network underlying plant responses to nutrient starvation by analyzing differentially expressed genes (DEGs) in *Arabidopsis* seedlings subjected to Pi starvation, as determined from previously published transcriptomic data (Wang et al., 2025). In contrast to normal Pi supply, Pi starvation resulted in a marked induction of numerous *PSR* genes, including *SPX1*, *SPX3*, *PS2*, *ISP1*, *AT4*, *PHT1;4*, *PHT1;5*, *PHT1;8*, and *PHT1;9* (**Figure 2A**). Previous research has demonstrated that these genes play a pivotal role in mediating the response of *Arabidopsis* to Pi starvation. Conversely, Pi starvation repressed the expression of well-characterized Fe starvation-responsive genes, such as *IRT1*, *FRO2*, *FIT*, *bHLH38*, *bHLH39*, *bHLH101*, *NAS1*, and *HMA3* (**Figure 2B**).

Additionally, we analyzed the expression levels of Pi starvation-responsive genes by RT-qPCR after an 8-day treatment of Pi starvation. The findings revealed that Pi starvation markedly induced the expression of *SPX1*, *SPX3*, *ISP1*, *PS2*, *PHT1;5*, and *PHT1;8* (**Figure 2C**), while significantly repressing the expression of *FIT*, *IRT1*, *FRO2*, *bHLH38*, and *bHLH39* (**Figure 2D**), consistent with the results obtained from transcriptomic sequencing. These expression analyses demonstrate that Pi starvation significantly enhances the expression of Pi starvation-responsive genes, such as *SPX1*, *SPX3*, *IPS1*, and *PS2*, while markedly suppressing the expression of genes associated with Fe starvation-responsive genes, including *FIT*, *IRT1*, *FRO2*, *bHLH38*, and *bHLH39*.

### 3.3 Transcription factor PHR1 enhances the promoter transcriptional activity of *SPX1*, *SPX2*, *SPX3*, *ISP1*, *PS2*, and *PHT1;4* genes

**Figure 3:**
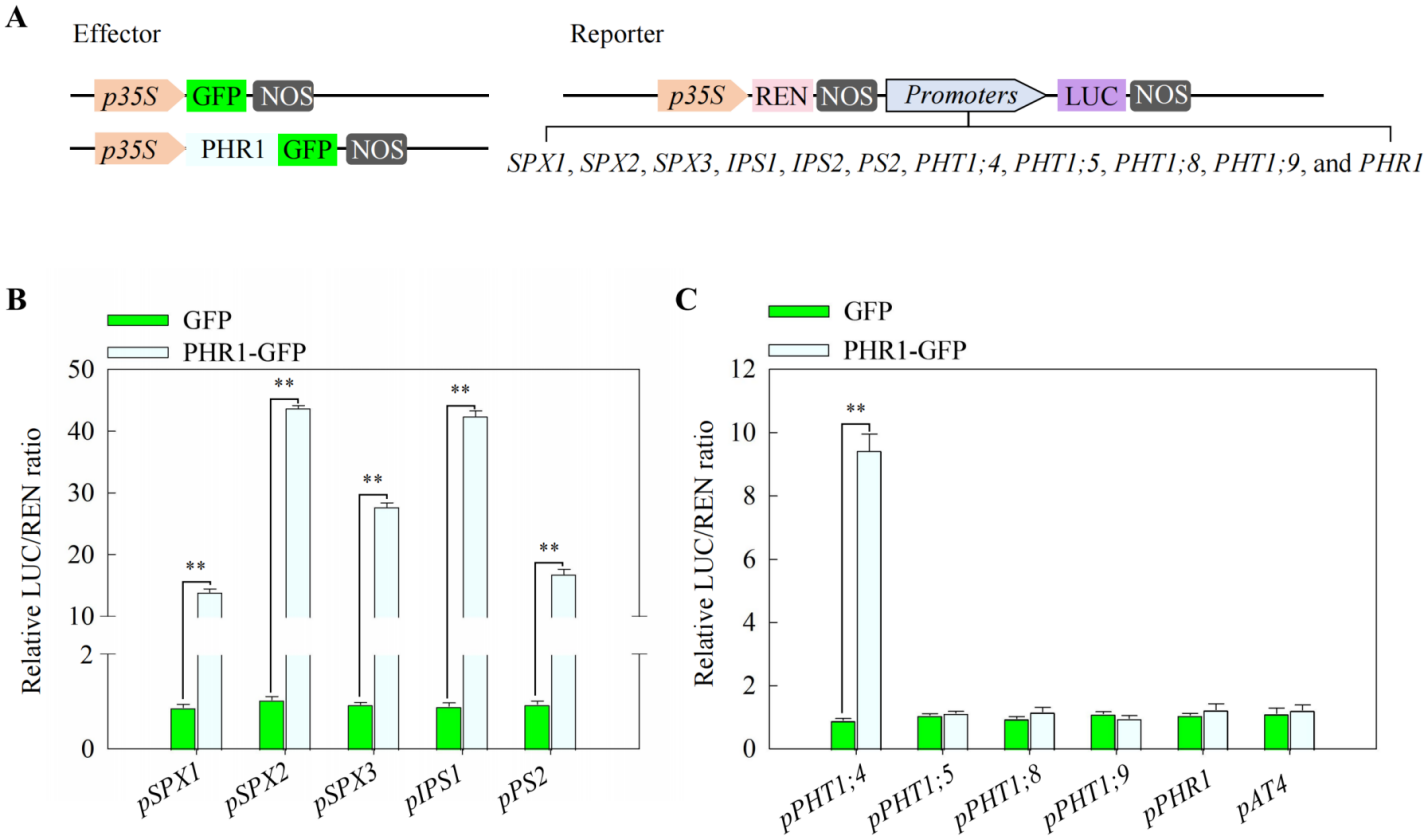
PHR1 enhances the promoter transcriptional activity of genes responsive to Pi starvation. **(A)** A schematic representation of the dual-luciferase effector and reporter constructs utilized in the mesophyll protoplast transient expression system. (*p35S*: *CaMV35S* promoter; GFP, green fluorescent protein; REN, Renilla luciferase; LUC, firefly luciferase). **(B)** The PHR1 protein enhances the promoter transcriptional activity of the Pi starvation-responsive genes *SPX1*, *SPX2*, *SPX3*, *IPS1*, and *PS2.* **(C)** The PHR1 protein also enhances the transcriptional activity of the *PHOSPHATE TRANSPORTER1;4* (*PHT1;4*) promoter, but transcriptional activity of *PHT1;5*, *PHT1;8*, *PHT1;9*, *PHR1*, and *AT4* genes promoter were not influenced by PHR1. Data are presented as the means ± SE with a sample size of *n* = 3. Asterisks denote statistically significant differences as determined by Student’s t-test (**P<0.01).

Extensive genetic, physiological, and biochemical experimental evidence has indicated that PHR1, the primary transcription factor involved in plant responses to Pi starvation, facilitates the expression of Pi starvation-responsive genes by directly binding to the PHR binding site (PIBS) *cis*-acting elements within their promoters, thus regulating Pi homeostasis (Sun et al., 2016; Wang et al., 2023; Mangalakkadan et al., 2026). In this study, we utilized the *Arabidopsis* mesophyll cell protoplast transient expression system, co-transfecting effector and reporter plasmids, to assess the influence of PHR1 on the transcriptional activity of the promoters of various *PSR* genes, including *SPX1*, *SPX2*, *SPX3*, *ISP1*, *PS2*, *PHT1;4*, *PHT1;5*, *PHT1;8*, *PHT1;9*, *PHR1*, and *AT4* (**Figure 3A**). The findings indicated that the PHR1-GFP fusion protein markedly increased the luciferase activity of the *LUC* reporter gene under the control of the *SPX1*, *SPX2*, *SPX3*, *ISP1*, *PS2*, and *PHT1;4* promoters, in comparison to the control protein GFP. Conversely, the luciferase activity of the *LUC* reporter gene driven by the promoters of *PHT1;5*, *PHT1;8*, *PHT1;9*, *PHR1*, and *AT4* remained unaffected (**Figures 3B, C**). These results suggest that PHR1 robustly transactivates the promoters of the *SPX1*, *SPX2*, *SPX3*, *ISP1, PS2*, and *PHT1;4* genes.

**Figure 4:**
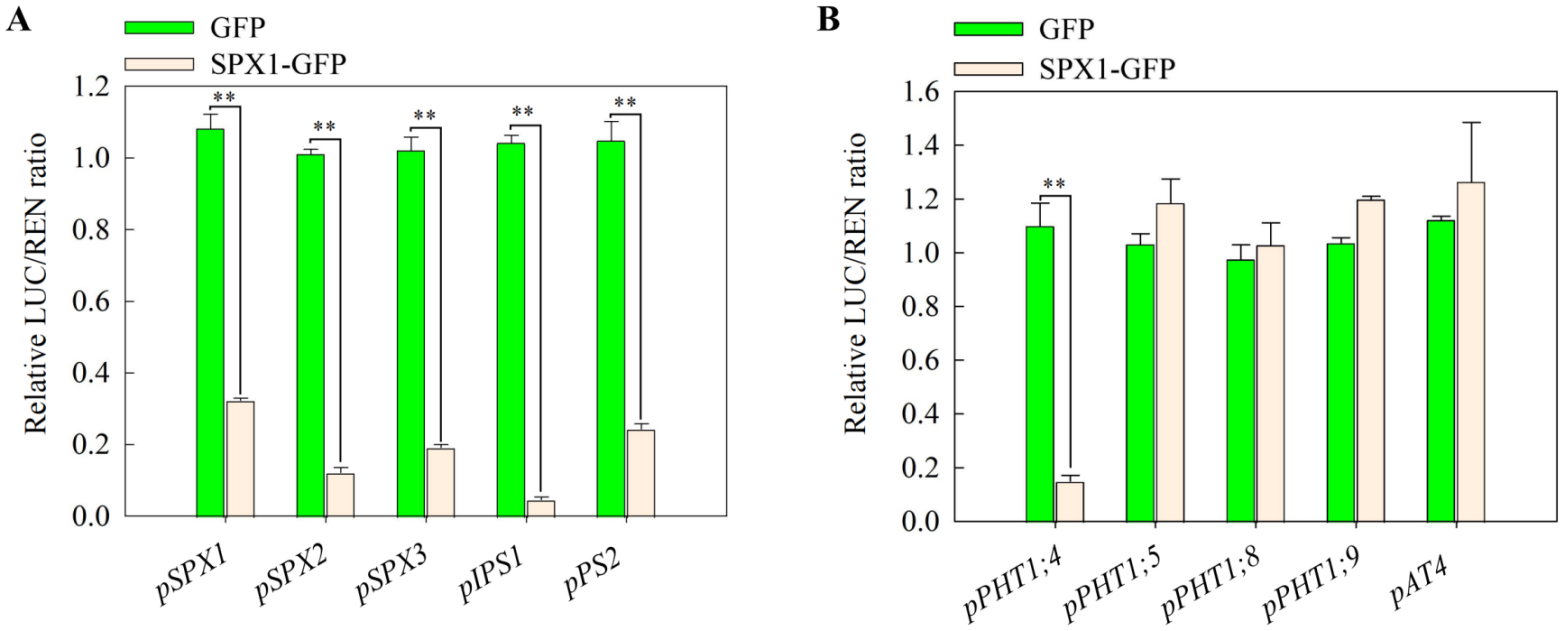
SPX1 suppresses the promoter transcriptional activity of genes responsive to Pi starvation. **(A)** The SPX1 protein suppresses the transcriptional activity of the promoters for the Pi starvation-responsive genes *SPX1*, *SPX2*, *SPX3*, *IPS1*, and *PS2.* **(B)** SPX1 protein also suppresses the transcriptional activity of the *PHOSPHATE TRANSPORTER1;4* (*PHT1;4*) promoter, but transcriptional activity of *PHT1;5*, *PHT1;8*, *PHT1;9*, and *AT4* genes promoter were not influenced by SPX1. Data are presented as the means ± SE with a sample size of *n* = 3.

Subsequently, we investigated the influence of the SPX1 protein on the transcriptional activity of gene promoters associated with Pi starvation. The findings indicated that, in comparison to the control protein GFP, the SPX1-GFP fusion protein significantly reduced the luciferase activity of the *LUC* reporter gene driven by the promoters of *SPX1*, *SPX2*, *SPX3*, *ISP1*, *PS2*, and *PHT1;4*. However, it did not alter the luciferase activity of the LUC reporter gene driven by the promoters of *PHT1;5*, *PHT1;8*, *PHT1;9*, and *AT4* (**Figures 4A, B**). Additionally, our results demonstrated that the SPX2 protein markedly inhibited the luciferase activity of the *LUC* reporter gene driven by the promoters of S*PX1*, *SPX2*, *SPX3*, *ISP1*, *PS2*, and *PHT1;4* (**Supplementary Figures 1A–C**). These findings suggest a functional redundancy between the SPX1 and SPX2 proteins in suppressing the expression of genes induced by Pi starvation. Overall, our research aligns closely with previous studies, underscoring the role of SPX1 and SPX2 as critical negative regulators in the plant response to Pi starvation.

### 3.4 Transcription factor FIT enhances the promoter transcriptional activity of *IRT1*, *FRO2*, and *NAS1* genes

**Figure 5:**
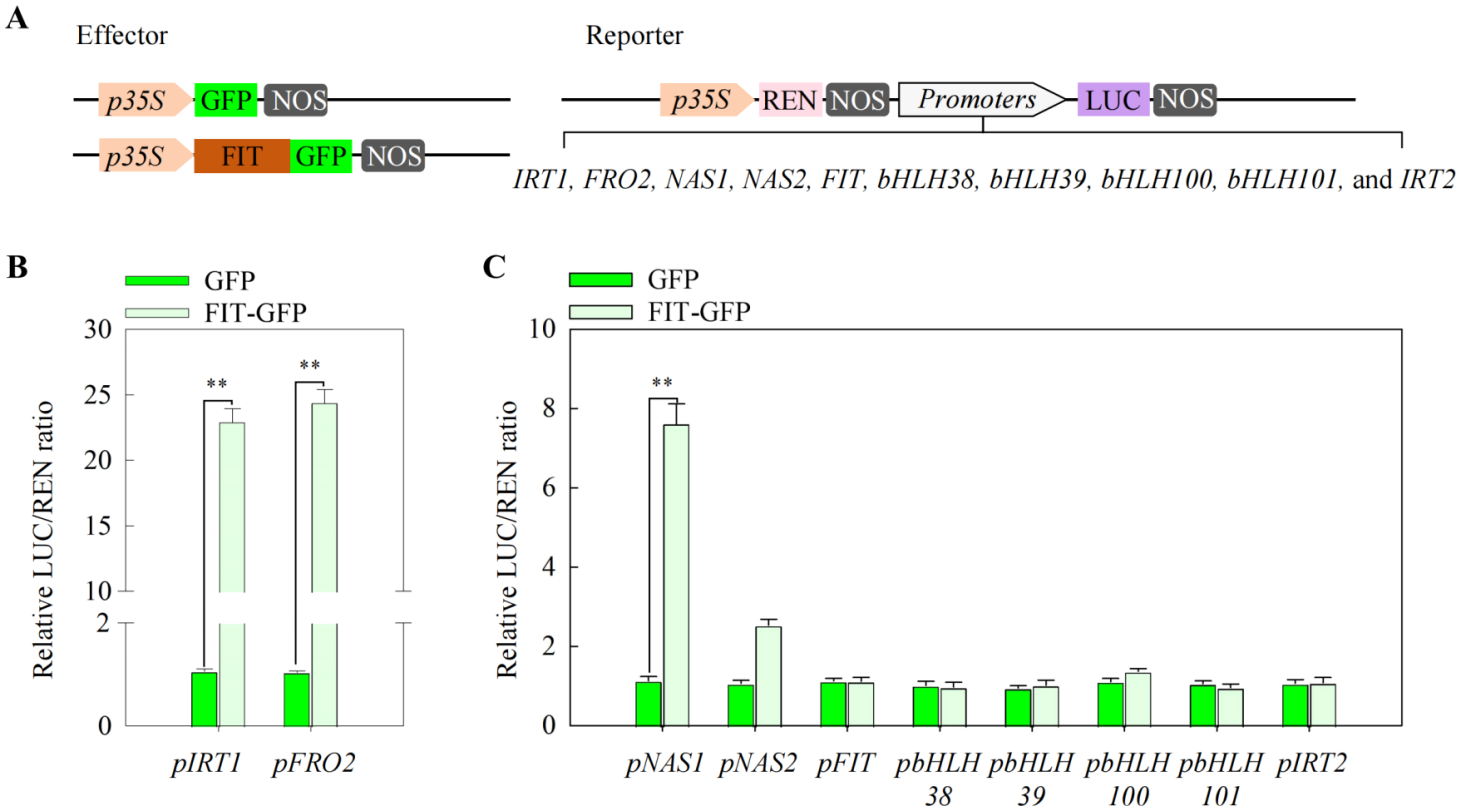
FIT augments the promoter transcriptional activity of genes responsive to Fe starvation. **(A)** A schematic illustration of the dual-luciferase effector and reporter constructs employed in the mesophyll protoplast transient expression system. **(B)** The FIT protein increases the promoter transcriptional activity of the Fe starvation-responsive genes *IRT1* and *FRO2.* **(C)** The FIT protein similarly enhances the transcriptional activity of the *NAS1* promoter, but transcriptional activity of *NAS2*, *FIT*, *bHLH38*, *bHLH39*, *bHLH100*, *bHLH101*, and *IRT2* genes promoter were not influenced by FIT. Data are presented as the means ± SE with a sample size of *n* = 3.

The *Arabidopsis* basic helix-loop-helix (bHLH) transcription factor FIT (FER-like iron deficiency-induced transcription factor) functions as a central regulator of the iron uptake system at the transcriptional level. It achieves this by forming heterodimers with other members of the bHLHIb subfamily, such as bHLH38/39/100/101, and directly binding to the promoter regions of essential genes involved in iron uptake, thereby activating the Fe absorption system (Cui et al., 2018). Herein, Pi starvation markedly suppressed the expression of genes related to Fe uptake. To further assess the potential application of the protoplast transient expression system within the genetic regulatory network governing plant Fe uptake, we investigated the influence of FIT on the transcriptional activity of the promoters of several Fe starvation-responsive genes, including *IRT1*, *FRO2*, *NAS1*, *NAS2*, *FIT*, *bHLH38*, *bHLH39*, *bHLH100*, *bHLH101*, and *IRT2*, as illustrated in **Figure 5A**. The results demonstrated that, in comparison to the control protein GFP, the FIT-GFP fusion protein markedly activated the *IRT1*, *FRO2*, and *NAS1* promoters, driving up to 20-fold and 22-fold increases in the luciferase activity for *IRT1* and *FRO2*, respectively, compared to the GFP control. Conversely, the luciferase activity of the *LUC* reporter gene driven by the promoters of *NAS2*, *FIT*, *bHLH38*, *bHLH39*, *bHLH100*, *bHLH101*, and *IRT2* remained unchanged (**Figures 5B, C**). These findings suggest that FIT substantially augments the transcriptional activity associated with the promoters of *IRT1*, *FRO2*, and *NAS1* genes. Previous research has unequivocally established that the ferric reductase FRO2 catalyzes the reduction of exogenous Fe^3+^ to Fe^2+^, which is subsequently taken up by the transporter *IRT1* into the roots. Both *FRO2* and *IRT1* are essential genes involved in the reduction strategy for Fe acquisition in dicotyledonous plants. In contrast, the transcription factor PHR1 did not influence the luciferase activity of the *LUC* reporter gene driven by the promoters of *FRO2* and *IRT1* (**Supplementary Figure 2**), nor did FIT affect the luciferase activity of the *LUC* reporter gene driven by the promoters of *SPX1*, *SPX2*, *SPX3*, *ISP1*, and *PS2* (**Supplementary Figure 3**). Collectively, our findings align closely with prior studies, thereby reinforcing the concept that the protoplast transient expression system serves as a rapid, reliable, and high-throughput methodology with significant potential for elucidating the genetic regulatory networks associated with plant nutrient starvation.

### 3.5 SPX1 suppresses the promoter transcriptional activation of the *PSI* genes *SPX1*, *SPX2*, *SPX3*, *IPS1*, *PS2*, and *PHT1;4* by PHR1

**Figure 6:**
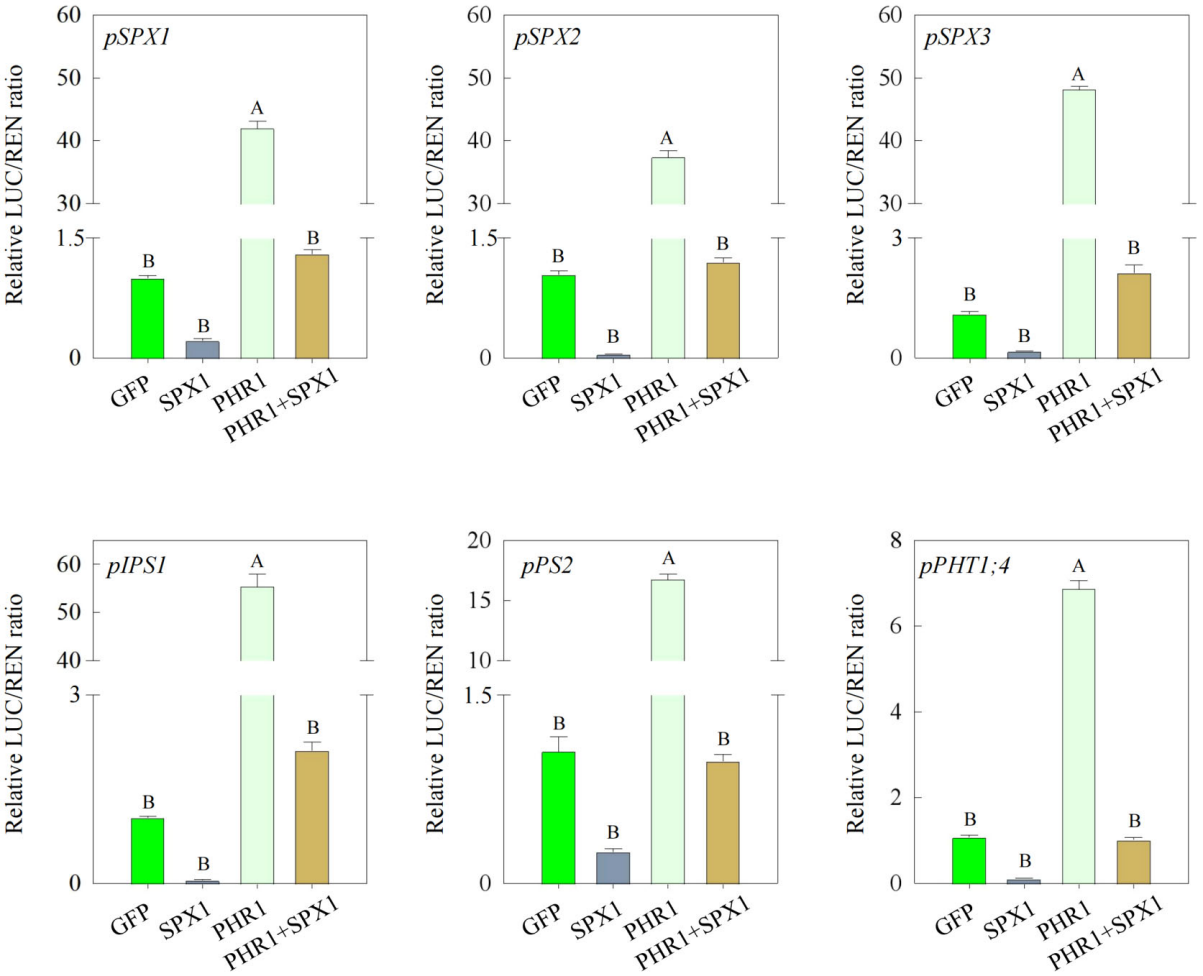
SPX1 protein suppresses PHR1-mediated the promoter transcriptional activity of genes responsive to Pi starvation. Data are presented as the means ± SE with a sample size of *n* = 3. Statistically significant differences between groups were evaluated using Tukey’s multiple comparisons test, with uppercase letters denoting significant differences (*P<0.01*).

The SPX1 protein acts as a crucial negative regulator in the transcriptional control of genes responsive to Pi starvation by interacting with PHR1 through its SPX domain. This interaction prevents PHR1 from binding to PIBS, a *cis*-acting element in the promoters of Pi starvation-responsive genes, thus lowering the expression of these genes and maintaining Pi balance in plants (Puga et al., 2014). To better understand the potential of the protoplast transient expression system in studying the combined or opposing regulation of downstream gene expression by multiple proteins, we analyzed the effects of expressing SPX1 and PHR1 proteins individually or together on the luciferase activity of the *LUC* reporter gene driven by the promoters of *PSI* genes, such as *SPX1*, *SPX2*, *SPX3*, *IPS1*, *PS2*, and *PHT1;4*. The findings revealed that, in comparison to the control protein GFP, the PHR1 protein significantly increased the luciferase activity of the *LUC* reporter gene driven by the promoters of *SPX1*, *SPX2*, *SPX3*, *IPS1*, *PS2*, and *PHT1;4*. When SPX1 and PHR1 proteins were co-expressed, the transcriptional activation effect of PHR1 on *PSI* gene promoters was inhibited (**Figure 6**). These findings indicate that the SPX1 protein negatively regulates the transcriptional activation of *PSI* genes mediated by the PHR1 protein. Therefore, the *Arabidopsis* mesophyll cell protoplast transient expression system serves as a rapid, reliable, and high-throughput research method with significant potential for elucidating genetic regulatory pathways and networks involved in plant responses to nutrient starvation, meriting further investigation and application.

## 4 Discussion

Transient expression systems, such as those employing *Arabidopsis* mesophyll cell protoplasts and *Nicotiana benthamiana* leaves, are extensively utilized in research examining interactions between transcription factors and promoters, analyzing promoter *cis*-acting element activity, investigating protein-protein interactions, and elucidating genetic regulatory pathways and networks (Yoo et al., 2007; Beritza et al., 2024). These systems have demonstrated significant potential for practical applications in the fields of plant growth, development, and stresses responses. However, their application in studies concerning plant nutrient starvation, particularly Pi and Fe starvation, remains underexplored. In this study, utilizing the *Arabidopsis* mesophyll cell protoplast transient expression system (**Figure 1**), we observed that PHR1 markedly enhanced the transcriptional activity of the promoters of *PSR* genes (Shin et al., 2004; Franco-Zorrilla et al., 2007; Sun et al., 2016) (**Figure 4**). Furthermore, the addition of the inhibitory protein SPX1 attenuated the activation effect of PHR1 (**Figure 6**). Furthermore, the core transcription factor associated with Fe starvation, FIT, significantly enhanced the transcriptional activity of the *IRT1* and *FRO2* promoters (**Figure 5B**), the crucial two genes for Fe starvation. These findings demonstrate that the protoplast transient expression system is an efficient and reliable method for investigating plant nutritional starvation responses. This system holds substantial potential for elucidating the genetic regulatory pathways and networks involved in both singular and synergistic nutritional starvation conditions.

To date, numerous studies employing techniques such as the construction of genetic lines have demonstrated that the SPX1-PHR1 module, a central regulatory component of plant responses to Pi starvation, modulates Pi homeostasis by regulating the expression of various *PSR* genes (Bustos et al., 2010; Puga et al., 2014). Transient expression assays conducted in *Arabidopsis* mesophyll cell protoplasts have demonstrated that PHR1 significantly enhances the transcriptional activity of *PSR* genes promoters (**Figure 4**). In contrast, the SPX1 protein suppresses the activation effect of PHR1 on its target genes (**Figure 6**). These findings indicate that the protoplast transient expression system is an effective tool for analyzing the genetic regulatory pathways and networks involved in plant responses to nutrient deprivation. Compared to traditional genetic methods, which are often characterized by lengthy experimental timelines, extensive spatial requirements, limited research targets, and low efficiency, our approach offers a high-throughput, rapid, and reliable alternative.

Additionally, FIT, a key transcription factor in plant responses to Fe starvation, regulates Fe homeostasis by directly controlling the expression of *IRT1* and *FRO2* (Wu and Ling, 2019; Gao and Dubos, 2021). Our study has further demonstrated, using this expedited and simplified method, that FIT significantly activates *IRT1* and *FRO2* expression (**Figure 5B**). Specifically, through the construction of effect and report vectors, this method facilitates direct quantitative analysis of the interactions between proteins and promoters, providing a cellular-level technical platform for analyzing the genetic regulatory networks of complex traits. This approach achieves efficient expression of target proteins and reporter genes within hours in a near-physiological cellular environment. This method outperforms traditional genetic analyses by offering a simplified workflow, rapid turnaround, high reproducibility, and scalability for high-throughput combinatorial screening. Furthermore, this technology holds potential for widespread application in the study of protein-protein interactions, post-translational modifications, and dynamic signal transduction processes. Notably, our findings underscore the growing importance of integrating high-throughput sequencing, quantitative PCR, and the protoplast transient expression system to rapidly identify key gene regulatory modules and pathways.

Under conditions of prolonged Pi deprivation, plants have developed intricate and precise transcriptional regulatory mechanisms to enhance the acquisition and utilization efficiency of Pi, thereby mitigating or overcoming Pi deficiency (Zulfiqar et al., 2025). In this study, transcriptomic and RT-qPCR analysis of wild-type (WT) plants subjected to Pi deprivation revealed a significant downregulation of genes associated with Fe starvation (**Figures 2B, D**). Previous research has established the core transcription factor FIT1 as essential for sustaining the Fe deficiency-induced expression of key uptake genes, such as *IRT1*, *FRO2*, and *AHA2*. Furthermore, during Pi deprivation, STOP1 activates the expression of *ALMT1*, leading to malate secretion, enhanced Fe accumulation, and the generation of reactive oxygen species (ROS), ultimately inhibiting primary root elongation in *Arabidopsis* (Balzergue et al., 2017; Mora-Macías et al., 2017). Our findings, in conjunction with prior studies, indicate that Fe absorption and accumulation are integral to plant responses to Pi deprivation, and that plants can ameliorate the toxic effects of excessive Fe accumulation by markedly suppressing the expression of the FIT-*FRO2/IRT1* module. Therefore, our study indicates that plants may regulate the homeostasis of Fe and Pi in roots via the FIT pathway. These findings offer novel insights into the genetic regulatory networks that underpin plant responses to multiple nutrient deficiencies. Nonetheless, addressing these challenges continues to encounter obstacles in several areas, including cellular & tissue heterogeneity, lack of *in situ* dynamic validation, non-linearity & multi-omics integration, and genetic redundancy & compensation.

Furthermore, while RT-qPCR and transcriptomic techniques have been extensively utilized to investigate differentially expressed genes across diverse tissues, including roots, leaves, and stems, and have produced significant findings, protoplast transient expression systems primarily employ leaves as the source material, especially in high-throughput studies of gene function. In our study, our findings align closely with established research in the domains of Pi-starvation and Fe-starvation, underscoring the stability and reliability of the mesophyll cell protoplast transient expression system. Despite various methods for preparing protoplasts from plant tissues like roots, these techniques face challenges such as difficult mass tissue collection, high costs, and inconsistent viability and transformation efficiency. These issues hinder their use in high-throughput gene research. In contrast, mesophyll cell protoplast preparation is simpler, cheaper, and more efficient, meeting the needs for quantity, viability, and transformation efficiency in experiments. This system not only facilitates the screening of results from transcriptome sequencing and RT-qPCR experiments but also identifies target genes for subsequent genetic experiments and the elucidation of genetic pathways.

Finally, while the mesophyll protoplast transient expression system offers distinct advantages, it is not without limitations. These include the limited viability of protoplasts, the significant influence of materials and procedures on transformation efficiency, and the difficulty in replicating regulatory effects observed at the tissue or whole-plant level. Consequently, this transient expression system is more appropriate for high-throughput analyses of molecular interactions among target genes and gene-gene modules. Future research should focus on optimizing protoplast preparation and transformation protocols, expanding their applicability to other crops, and systematically elucidating the genetic regulatory network involved in crop nutrient starvation responses. This can be achieved by integrating single-cell sequencing with protein interaction omics because they have been successfully applied to various tissues in multiple species. During this process, mesophyll protoplasts can act as a versatile system for investigating plant cell reprogramming from single cell to plants (Pasternak et al., 2020). Besides, future research directions should more precisely focus on the selective preparation of protoplasts from different plant tissues (such as leaf, root, seed, and SAM) and their targeted application in gene function validation. In addition, plants often face simultaneous deficiencies or excesses of multiple nutrients, rather than a single nutrient stress. Using a protoplast system, it is possible to systematically study the mechanisms of signal network interactions under multiple nutrient stresses. Elias et al. (2023) have demonstrated a DNA-free CRISPR-Cas9 gene editing system in papaya protoplasts on multiple target genes for use in papaya crop improvement. Hence, protoplast-based functional genomics and genome editing can contribute to address public concerns over transgenic technologies while enabling the rapid development of stress-tolerant cultivars (Hsieh et al., 2026).

## 5 Conclusions

Our research has demonstrated that the *Arabidopsis* mesophyll cell protoplast transient expression system possesses extensive potential for investigating Pi and Fe starvation. Analysis of promoter activity indicated that PHR1 substantially enhances the transcriptional activity of *PSR* promoters, whereas the SPX1 protein suppresses PHR1-mediated activation of these promoters. Furthermore, FIT significantly enhances the transcriptional activity of *IRT1* and *FRO2* promoters. This study advances our comprehension of the core regulatory modules PHR-SPX1 (pertaining to Pi starvation) and FIT-*FRO2/IRT1* (pertaining to Fe starvation), along with their associated genetic regulatory pathways. Additionally, it underscores the considerable advantages of the protoplast transient expression system in elucidating the genetic regulatory networks involved in plant responses to nutrient starvation, characterized by its rapidity, reliability, and high-throughput capabilities. Hence, these findings provide a novel and robust foundation supporting the large-scale application of this technology.

## Data Availability

The original contributions presented in the study are included in the article/**Supplementary Material**. Further inquiries can be directed to the corresponding author.
