# Supplementary material for: Mesophyll protoplasts: a key tool for deciphering plant nutrient starvation pathways
Source: Front Plant Sci. 2026 Jul 23;17:1825102. doi: 10.3389/fpls.2026.1825102 (PMC13441832; doi:10.3389/fpls.2026.1825102)
Supplement: Supplementary file 1 [file DataSheet1.pdf]

Supplementary Data

Supplementary Figures

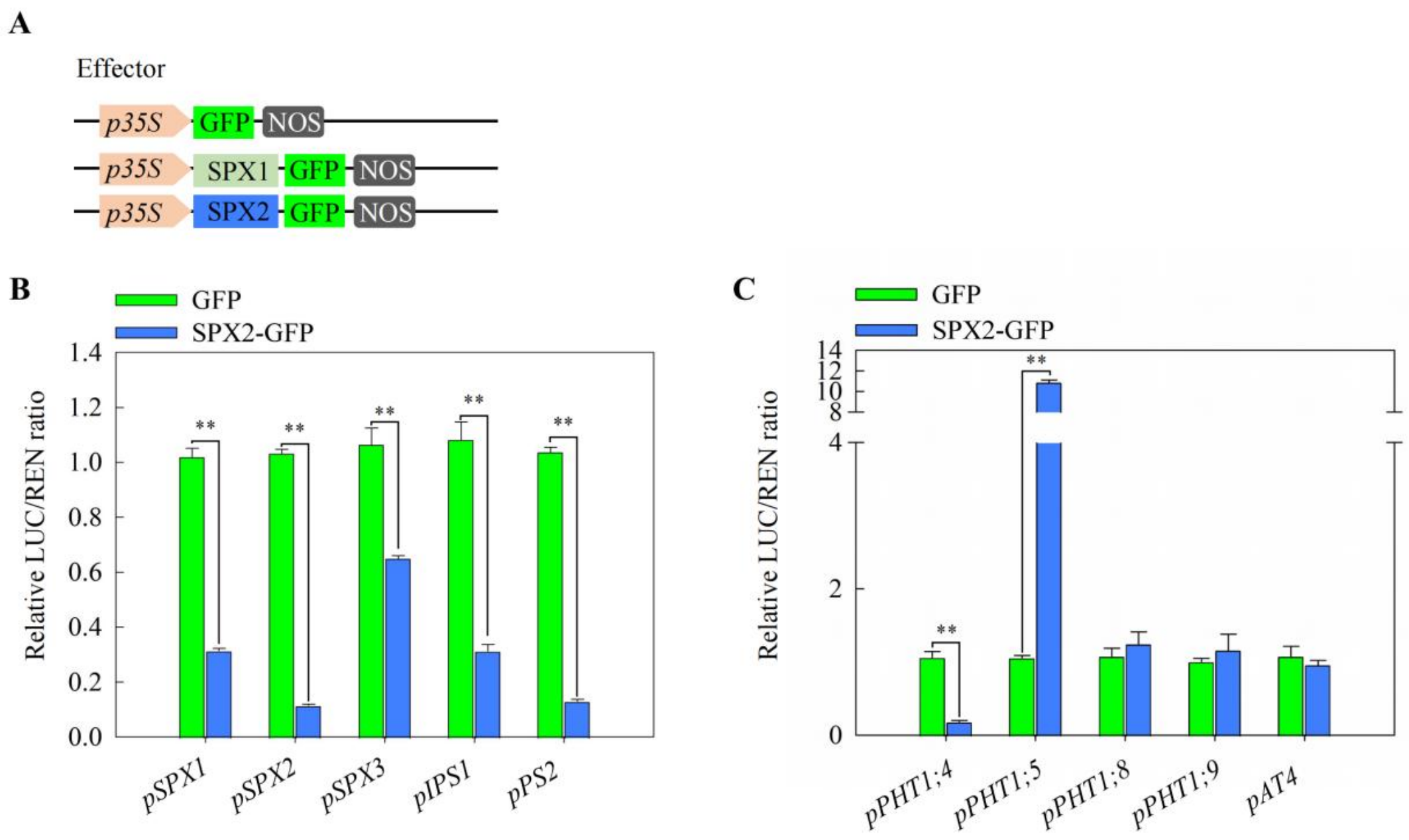

Supplementary Figure 1. SPX2 inhibits the promoter transcriptional activity of genes responsive to Pi starvation.

(**A**) A schematic representation of effector constructs *pCaMV35S-SPX1-GFP* and *pCaMV35S-SPX2-GFP* in transient dual-luciferase assays.

(**B**) The SPX2 protein inhibits the transcriptional activity of the promoters for the Pi starvation-responsive genes *SPX1*, *SPX2*, *SPX3*, *IPS1*, and *PS2*.

(**C**) SPX2 protein also suppresses the transcriptional activity of the *PHOSPHATE TRANSPORTER1;4* (*PHT1;4*) promoter, but enhances the promoter transcriptional activity of *PHT1;5* gene, while does not influence promoters activity of *PHT1;8*, *PHT1;9*, and *AT4*.

Data are presented as the mean ± standard error (n = 3).

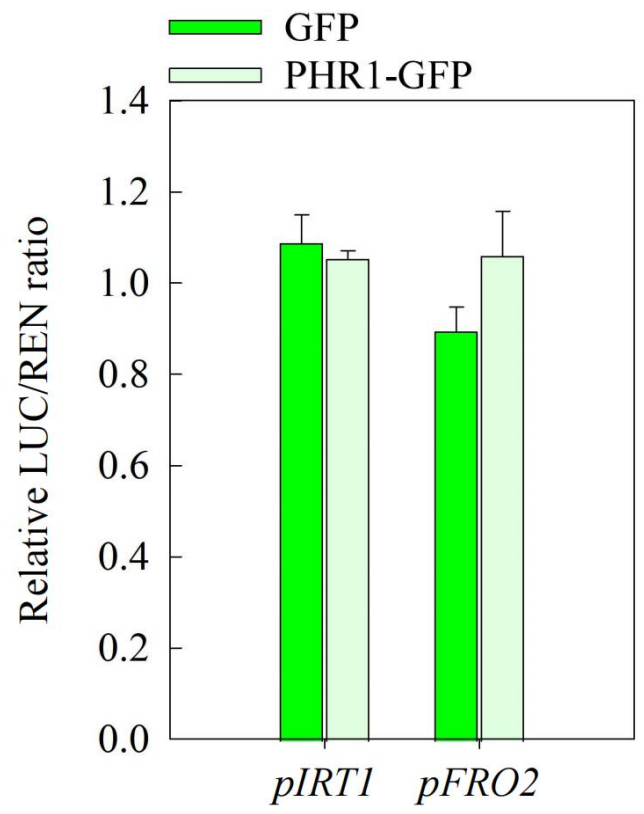

Supplementary Figure 2. PHR1 does not affect the promoter transcriptional activity of the *IRT1* and *FRO2* genes.

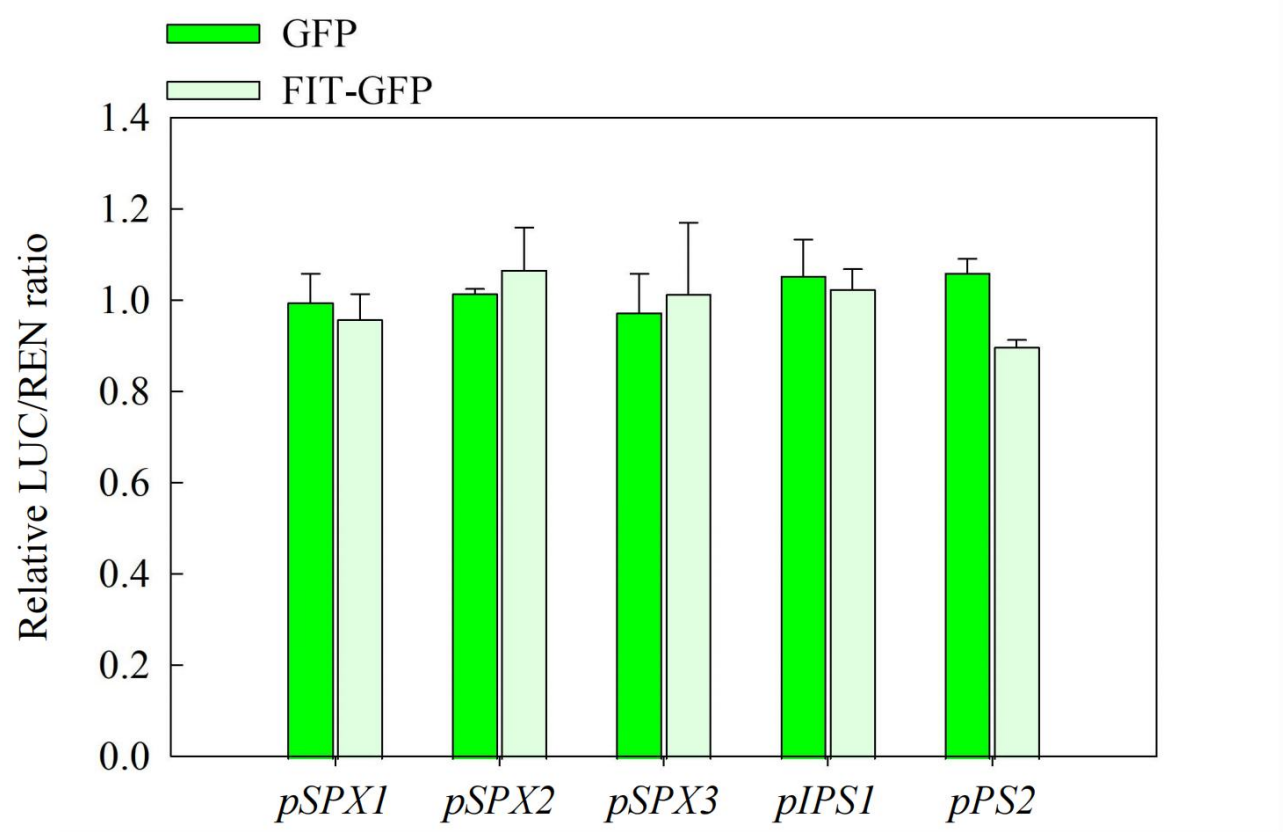

Supplementary Figure 3. FIT does not influence the promoter transcriptional activity of the *SPX1*, *SPX2*, *SPX3*, *IPS1*, and *PS2* genes.
