# Supplementary material for: Mesophyll protoplasts: a key tool for deciphering plant nutrient starvation pathways
Source: Front Plant Sci. 2026 Jul 23;17:1825102. doi: 10.3389/fpls.2026.1825102 (PMC13441832; doi:10.3389/fpls.2026.1825102)
Supplement: Supplementary file 2 [file DataSheet2.pdf]

Supplementary Table

Supplementary Table S1 The primers used in this study

Primer Name	Forward primer (5′-3′)	Reverse primer(5′-3′)
**Primers used for LUC/REN ration**		
pCaMV35S-SPX1-GFP	gacaagcttggtacctctagaATGAAGTTTGGTAAGAGTCT	gcccttgctcaccatactagtTTTGGCTTCTTGCTCCAACAAT
pCaMV35S-SPX2-GFP	gacaagcttggtacctctagaATGAAGTTCGGCAAGAGCCT	gcccttgctcaccatactagtTTTCGCTACTTGTTCAAGGAC
pCaMV35S-PHR1-GFP	gacaagcttggtacctctagaATGGAGGCTCGTCCAGTTCA	gcccttgctcaccatactagtATTATCGATTTTGGGACGC
pCaMV35S-FIT-GFP	gacaagcttggtacctctagaATGGAAGGAAGAGTCAACGC	gcccttgctcaccatactagtAGTAAATGACTTGATGAATTCAAA
pSPX1:LUC	gtcgacggtatcgataagcttGATAAAAAAGTTTTCTGCTC	cgctctagaactagtggatccAGCTCTTTTATTTTCTGG
pSPX2:LUC	gtcgacggtatcgataagcttTTTCTTGAAAGAGAAGTAAT	cgctctagaactagtggatccGGTTAGCTGAGAATAAGGAA
pSPX3:LUC	gtcgacggtatcgataagcttAAAAGAGAAGAGAAACAAACTTTATTGTT	cgctctagaactagtggatccCAAAATATAAACTCAAGTTTTTACCTTAAAA
pISP1:LUC	gtcgacggtatcgataagcttAACTAGGTGGATCCAGTCTA	cgctctagaactagtggatccTCATTGCTCCATATCTTAAA
pPS2:LUC	gtcgacggtatcgataagcttGAAAGTATGGAAAAAAAAAAAAGTGTG	cgctctagaactagtggatccGACTCAGGAAATTTCTAAAGAAATTAGTG
pPHT1;4:LUC	gtcgacggtatcgataagcttACAGCTATTTTTTTGGATATTAAAAGAAT	cgctctagaactagtggatccTCTTCTTCTCCTCTGCAATTTTTCA
pPHT1;5:LUC	gtcgacggtatcgataagcttTCGTGGTATTCAAAGAAATAAAGATCT	cgctctagaactagtggatccTGTTCCGGCGGATTTTGA
pPHT1;8:LUC	gtcgacggtatcgataagcttCTGGTGAGCTTCAAGAAACGTG	cgctctagaactagtggatccCGTTGGTTTTTTTATCTGAGATTCC
pPHT1;9:LUC	gtcgacggtatcgataagcttTATGAAACTCATCTTCCACATTTAGTTTC	cgctctagaactagtggatccGTTTCGTCGATTTTTTAAGAATTT
pPHR1:LUC	gtcgacggtatcgataagcttCAAAGTCGGAAAAGCTTTGCC	cgctctagaactagtggatccTGTTGTCCTGCAAGAGAGAATCA
pAT4:LUC	gtcgacggtatcgataagcttGGAACAGGTTCAAGATGTTC	cgctctagaactagtggatccATTGATAATTATGATATTGG
pIRT1:LUC	gtcgacggtatcgataagcttAATTTGTGTCAATATCTGATTTGGT	cgctctagaactagtggatccTTTTTTTTTTTTTCTTTTTTCTTTTG
pFRO2:LUC	gtcgacggtatcgataagcttCACGTGTATTTTACACACGTACGTA	cgctctagaactagtggatccCCTCTCTTTCCTCTCAGGATTTCTA
pNAS1:LUC	gtcgacggtatcgataagcttAAGCAAACGAAACAATC	cgctctagaactagtggatccATCGACACTATGAGGTTA
pNAS2:LUC	gtcgacggtatcgataagcttGATTCGGATATTATAGC	cgctctagaactagtggatccGTCGACACTATGAAGAGAG
pFIT:LUC	gtcgacggtatcgataagcttAGAAACATTACAGACCGAAATTT	cgctctagaactagtggatccTTGTGTGTTTTTGTCAATGAAGAA

pbHLH38:LUC	gtcgacggtatcgataagcttTTTATAGTTATGTTATGTCGTCCTT	cgctctagaactagtggatccTTTTTGCTTAATCAAGGACAAGG
pbHLH39:LUC	gtcgacggtatcgataagcttTTCGAAGAAGCAACCTTCTCTGT	cgctctagaactagtggatccTTTTGCTTACTAAGGACAAGTATT
pbHLH100:LUC	gtcgacggtatcgataagcttCTACGTTCTAGACGTTCAGTAAAG	cgctctagaactagtggatccTTTGAGTTTTAGATAGTTACTCTGTT
pbHLH101:LUC	gtcgacggtatcgataagcttCTCACTAGCTAATTCGTGGATATTT	cgctctagaactagtggatccTTTTTTTGTCCACTTATGTTATGTGT
pIRT2:LUC	gtcgacggtatcgataagcttTTCTTAACTTAGCAACACAC	cgctctagaactagtggatccAGTATTGAGATTGTTTTAT
**Primers used for RT-qPCR**		
qUBQ10	TCCTCAGGCTCCGTGGTGGT	AGCCTCTGCTGGTCCGGAGG
qSPX1	TCCCTGCTAACGAAACTGAGTCTGA	AATAGAGCGAGTGTGCCTCTGTCT
qSPX3	CCGCCGCTAAATATCTCCGATT	TGTCCGAAGGAAAGTGACAACA
qIPS1	AGACTGCAGAAGGCTGATTCAGA	TTGCCCAATTTCTAGAGGGAGA
qPS2	TATTGCCTCGTGCTCTTCGTGTG	TGAGGAAGAAGAAACACCACAGTTG
qPHT1;5	CGGAGATACGGTGGTGGAGAT	CCAATTCACAGAGTAATTTGGTTGC
qPHT1;8	ATGGCTGTTTGCGTGGCGTTTA	CGTCGTGGCTTCGTCGGAGATA
qFIT1	ACCTGAAGCTGTGGATCACTGGAT	TTGACTCACGTTAGAGCAAGCGTTA
qIRT1	TTCTCGGGTGCGGTGGAATG	CCGATAGAGAATCGAGACGTAAACC
qFRO2	TGGAAGGTTCGAGTGTGGGAGT	GGTCAAATCACAGGGCCACCTT
qbHLH38	TGGGATAGAAGAAGATGGGTTTGTT	TTGGTCCAGATCAGTGTTAGATTCA
qbHLH39	TGCGAAGAGTTAAGTCAGAGGATGT	ACTCGACTACAACGCTTTGTCAAGA
